# Comparison of the Effect of Oral Versus Intravenous Bisphosphonate Administration on Osteoclastogenesis in Advanced-Stage Medication-Related Osteonecrosis of the Jaw Patients

**DOI:** 10.3390/jcm10132988

**Published:** 2021-07-04

**Authors:** Hye-Won Kim, Min-Woo Lee, Jung-Hwan Lee, Moon-Young Kim

**Affiliations:** 1 Department of Oral and Maxillofacial Surgery, College of Dentistry, Dankook University, 119 Dandae-ro, Cheonan 31116, Korea; kidbethel11@gmail.com (H.-W.K.); lmw920@daum.net (M.-W.L.); 2 Institute of Tissue Regeneration Engineering (ITREN), Dankook University, Cheonan 31116, Korea; 3 UCL Eastman-Korea Dental Medicine Innovation Centre, Dankook University, Cheonan 31116, Korea; 4 Department of Nanobiomedical Science & BK21 PLUS NBM Global Research Center for Regenerative Medicine, Dankook University, Chungnam, Cheonan 31116, Korea; 5 Department of Biomaterials Science, College of Dentistry, Dankook University, Chungnam, Cheonan 31116, Korea

**Keywords:** osteonecrosis, medication-associated osteonecrosis of the jaw, osteoclasts, biomarkers, potassium channels

## Abstract

It is yet unknown whether the intravenous administration route alone can fully account for the exacerbation of medication-related osteonecrosis of the jaw (MRONJ). The purpose of this retrospective study was to identify the potential role of the bisphosphonate (BP) administration route as an independent prognostic factor for non-cancerous, stage III MRONJ patients. Bone samples were retrospectively obtained from two groups of osteoporosis patients who underwent surgery for the treatment of stage III MRONJ. Among the subjects, 10 had a history of only oral BP consumption and 10 of intravenous (IV) BP administration. The samples were assessed for osteoclast morphology and immunohistochemical expression of the receptor activator of NF-κB ligand (RANKL), osteoprotegerin (OPG), and potassium calcium-activated channel subfamily N member 4 (Kcnn4). Although the osteoclasts derived from both groups exhibited no significant differences in the mean quantity, diameter, and nuclearity, significantly attenuated tartrate-resistant acid phosphatase activity was noted among the IV BP-induced MRONJ bones compared to those of the oral BP group. Significant suppression of the RANKL/OPG ratio and Kcnn4 expression among the retrieved bones of IV BP group patients was also noted. Our results indicate the potential of the BP administration route as an independent prognostic factor for advanced-stage MRONJ, regardless of the dosage or indication for which the BP was prescribed.

## 1. Introduction

Medication-related osteonecrosis of the jaw (MRONJ) has emerged as a devastating side effect induced secondary to long-term administration of antiresorptive medications since its first description in 2003 [1,2,3,4]. It is characterized by the features of an open alveolar socket, exposed necrotic bone or sequestra, increased expression of inflammatory infiltrates, osseous sclerosis, and radiologic signs of osteolysis [5]. Despite the importance of the disease, the management of MRONJ has been hampered by the lack of a well-defined standardized set of prognostic indicators. The complex issue related to the unfathomed complex pathophysiology of this disease has in fact led to the establishment of several fundamental milestone theories and subsequent novel treatment strategies for MRONJ patients. Owing to these innovative techniques, it is now expected that effective downstaging and management of MRONJ from its earliest stages to the most advanced cases, even for those with concomitant osteo-metabolic diseases, could be possible [6,7,8,9]. However, no specific biomarker for MRONJ has been established in clinical trials to date.

The pathogenesis of MRONJ is not currently correlated with one specific etiological mechanism [1,4], but it is speculated that the inhibitory pharmacological effect on osteoclasts by antiresorptive drugs such as bisphosphonates (BP) and denosumab plays a central role [4,10,11]. Bisphosphonates represent a major class of antiresorptive medications prescribed for the treatment and prevention of osteoporosis, multiple myeloma, and skeletal related events (SREs) of malignant solid tumors [1,12]. It lowers bone turnover following oral surgery via the inhibition of osteoclast-mediated bone resorption, which occurs through the disruption of the mechanisms crucial for the formation, proliferation, and resorption capability of osteoclasts [2,4,11,13,14,15,16].

During the past two decades, our knowledge of the molecular mechanisms that regulate osteoclastogenic activity has been greatly advanced through the establishment of osteoclast-specific bone metabolism markers via various in vitro and in vivo models [17,18,19,20,21,22,23]. In particular, the discovery of the signaling system involving the receptor activator of NF-κB ligand (RANKL), its receptor RANK, and osteoprotegerin (OPG), the decoy receptor of RANKL, has established a fundamental milestone in understanding bone physiology [20,24,25]. The RANKL/OPG ratio is considered to play a pivotal role in determining osteoclastogenic activity and bone mass [4,11,24,25,26]. Its deficiency following BP treatment has also been correlated with the development of MRONJ in several previous studies [4,11,27].

Another novel protein that has recently emerged as a promising biomarker for the evaluation of RANKL-induced osteoclast activity is Kcnn4 [17,28], one of the four constituent proteins of the potassium calcium-activated channel subfamily N (Kcnn) family that form a voltage-independent potassium channel [29]. This protein encodes a channel for intracellular Ca^2+^ influx and has great clinical significance in various cell physiological activities [15,30,31]. Investigation into the Kcnn4 function has already revealed its participation in the invasion and metastasis of several malignant conditions such as papillary thyroid [29], pancreatic [32], breast [33], hepatocellular [30,34], clear cell renal cell [35], lung [36], endometrial [37], and human prostate [38] carcinoma. However, although the previous data are suggestive, formal clinical investigation of the role of Kcnn4 in the crosstalk between bisphosphonate administration and the pathogenesis of MRONJ is lacking.

Due to its multifactorial etiology, the prevalence, severity, and prognosis of MRONJ are influenced by various factors [3,4,39]. Recent studies have also assessed the regulatory effect of BP administration on the osteogenic potential and phenotype of oral mesenchymal stem cells [40,41,42,43]. However, to date, limited studies investigating the potential effect of the route of BP administration on MRONJ prognosis have been performed. Many authors have demonstrated increased prevalence [9,12,44,45] and severity [12,46,47,48,49,50] of MRONJ following the intravenous (IV) administration of antiresorptive medications compared to those of patients with history of only oral BP consumption. Nonetheless, owing to the differences in the indications for which IV and oral BPs were prescribed in the previous trials [9,12,45,46,47,48,49,50,51], it is yet unknown whether the IV route alone can fully account for exacerbated MRONJ prognosis, or whether the previous findings were due to the larger doses of BPs prescribed for the intravenous group.

Herein, we identify the administration route of bisphosphonates as an independent prognostic factor for MRONJ. The tartrate-resistant acid phosphatase (TRAP)-positive osteoclasts, RANKL/OPG ratios, and Kcnn4 levels of non-cancerous patients with MRONJ induced by equal doses of intravenous and oral bisphosphonates are compared.

## 2. Materials and Methods

### 2.1. Patient Selection and Specimen Harvesting

After approval from the institutional review board of Dankook University Dental Hospital (IRB number, DKUDH IRB 2020–09-006), retrospective analyses were performed for routine jaw bone specimens obtained from 20 patients who underwent surgery for the treatment of clinically and histologically confirmed MRONJ of the mandible at Dankook University Hospital from 2017 to 2020. The 20 patients were divided into two groups according to the route of BP administration: (1) 10 with history of only oral bisphosphonate consumption (Group 1; Oral-BP Group) and (2) 10 with treatment history of intravenous bisphosphonates (Group 2; IV-BP Group).

The exclusion criteria for this study were patients classified as stages other than stage III (according to the MRONJ staging system previously described in the 2014 position paper published by the American Association of Oral and Maxillofacial Surgeons [39]), patients for whom the presence of necrotic bone was not confirmed via surgical biopsy, patients with a history of bisphosphonate or denosumab consumption for the management of conditions other than osteoporosis, and those documented to suffer from medical conditions that are known to affect bone regeneration such as anemia, diabetes, and a history of prolonged steroid treatment. The descriptive data of the subjects included for this study are summarized in Table 1.

### 2.2. Histochemistry

Immediately after retrieval, the bone specimens were fixed in 4% paraformaldehyde in 0.1 M phosphate buffered solution (pH 7.4) for 48 h and incubated for 12 h in HCl (Calci-Clear Rapid, National Diagnostics, Atlanta, GA, USA) for decalcification. The specimens were then embedded in paraffin and sliced in sections of 6-μm thickness using a rotary microtome (Leica Microsystems, Wetzlar, Germany). The histological sections were mounted on glass slides and stained with hematoxylin–eosin (H&E) to visualize the typical morphological features of osteoclasts.

### 2.3. Immunohistochemistry

Immunohistochemical (IHC) assays for detection of RANKL, OPG, and Kcnn4 were performed on the Ventana Discovery-Ultra IHC in situ hybridization automated staining platform (Ventana Medical Systems Inc., Tucson, AZ, USA) according to the manufacturer’s recommendations. After the specimens were fixed, decalcified, and routinely embedded in paraffin as described above, 6-μm paraffin-embedded sections were placed on glass slides coated with 2% 3-aminopropyltriethylsilane (Sigma Chemicals, St. Louis, MO, USA). Slides were then deparaffinized in EZ Prep (950–102; Ventana Medical Systems, Tucson, AZ, USA) and treated with Ventana Cell Conditioning Solution (760–107; Ventana Medical Systems, Tucson, AZ, USA) for 32 min at 95 °C to achieve antigen retrieval. Sections were incubated at 37 °C for 4 h with the following primary antibodies: anti-RANKL (PA5-20291, Invitrogen, Carlsbad, CA, USA), diluted 1:1600; anti-OPG (PA5-86053, Invitrogen, Carlsbad, CA, USA), diluted 1:400; and anti-Kcnn4 (PA5-33875, Invitrogen, Carlsbad, CA, USA), diluted 1:400. Slides were developed via 16 min incubation at 37 °C with ready-to-use Omnimap anti-Rabbit HRP solution (760-4311; Ventana Medical Systems, Tucson, AZ, USA) at the provided pre-diluted concentration, as a secondary antibody, and peroxidase activity was visualized by immersing the specimens in 3,3-diaminobenzidine (DAB) substrate (Cat#760-4311, Ventana Roche OmniMap). Finally, to visualize the nuclei, slides were counterstained with Ventana Hematoxylin reagent and mounted as in routine processing.

### 2.4. TRAP Staining

For TRAP staining of osteoclasts, the retrieved bone specimens were embedded in paraffin and sliced in sections of 6-μm thickness using a rotary microtome (Leica Microsystems, Wetzlar, Germany) without prior calcification. Following deparaffinization and rehydration, the slides were incubated at 37 °C for 5 h using a TRAP detection system (TRACP & ALP double-stain kit, MK 300, TakaraBio, Kasatsu, Japan) through which an azoic (purplish red) dye is generated in the presence of the enzyme, according to the manufacturer’s instructions. Slides were then rinsed in distilled water, counterstained with Ventana Hematoxylin reagent, air-dried, and mounted.

### 2.5. Quantitative and Qualitative Analysis

All stained slides were digitized via the Pannoramic 250 Flash III Slide Scanner (3DHISTECH Kft., Budapest, Hungary) after they had been quality-checked under a bright-field microscope (BX-41, Olympus Optical, Tokyo, Japan). Two visual fields with a high probability for the presence of osteoclasts, such as the subperiosteal bone, bone trabeculae, endosteal structures, and connective tissues directly adjacent to the bone, were selected for each slide. A region of interest (ROI) was defined as the non-bony medullary tissues within each visual field. All qualitative and quantitative analyses were performed only within the ROIs.

The slides from all groups were anonymized and the total number, diameter, and number of nuclei were quantified for the osteoclasts observed within each ROI. All histomorphometric analysis was independently performed by two calibrated investigators (H.K. and M.L.) using the Pannoramic Viewer version 2.1 (3DHISTECH) under 800× magnification. The observers were blinded to the scores of other markers as well as to clinical information regarding patient data. The following four criteria adapted from Gross et al. [2] were used to define osteoclasts: (1) presence of at least two nuclei; (2) size of cell body larger than those of two fused mononuclear cells; (3) proximity to bone; (4) absence of nearby granulomatous foci or foreign particles. If the inter-individual differences for each score exceeded 10% the corresponding slides were reassessed to reach a consensus. Results were expressed as osteoclast number per ROI, mean osteoclast diameter per osteoclast, and mean number of nuclei per osteoclast.

For the quantitative analysis of the number of RANKL, OPG, Kcnn4, and TRAP positive cells, the magnified (800×) stained images were loaded on the Image Pro Plus 7 software (Media Cybernetics, Bethesda, MD, USA). The software was trained to identify positively stained cells for each immunohistochemical marker, and all slides were batched and analyzed using the same algorithm. The positive cells identified via this algorithm were manually counted by two independent observers as described above. For TRAP-positive multinucleated cells (TRAP+ MNCs), only cells containing more than three nuclei were counted as mature osteoclasts.

### 2.6. Statistical Analysis

Statistical analysis was performed using the SPSS 27.0 software (SPSS Inc., Chicago, IL, USA). The measures of key outcomes were expressed as means ± standard deviation (SD). Normality of distribution was assessed by the Kolmogorov–Smirnov test, and the means of outcomes between the two groups were compared using the Mann–Whitney U test. A two-sided *p*-value of less than 0.05 was considered statistically significant.

## 3. Results

### 3.1. Characteristics of the Included Subjects

The descriptive data of the patients included for this study is summarized in Table 1*,* as mentioned above. There was no significant difference in the mean age of both groups (*p* = 0.631).

### 3.2. Quantitative and Qualitative Analysis of Osteoclasts

**Figure 1:**
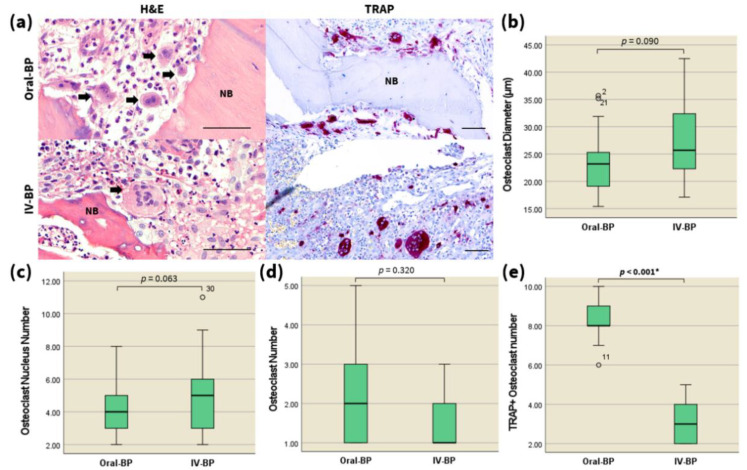
(**a**) High-magnification images (800×) of H&E (left) and TRAP (right) staining exhibiting round-shaped, multinucleated, giant osteoclasts (black arrows) at the necrotic jaw bone samples retrieved from IV-BP and oral-BP induced MRONJ subjects. Scale bars = 50 μm. Statistical analysis of the mean osteoclast diameter (**b**), number of nuclei (**c**), total osteoclast number (**d**), and TRAP-positive osteoclasts (**e**) in the necrotic bone specimen from (**a**), via the Mann–Whitney U test (*n* = 20 per group). ° marks statistical outliers. For detailed data, see Table 2. NB, necrotic bone; BP, bisphosphonates; IV, intravenous. * *p* < 0.001.

To investigate the effect of the route of BP administration on osteoclasts, digitized images of the stained pathologic sections derived from the subjects of the two distinct groups were analyzed using the Pannoramic Viewer software. The osteoclasts observed among both MRONJ groups were round-shaped, giant in size, multinucleated, and showed lack of a ruffled border adjacent to the bone surface. The nuclei of these osteoclasts also often appeared to be pyknotic. Subjects in the IV-BP group featured osteoclasts with a slightly larger diameter and more nuclei than those of oral-BP subjects, but no significant differences could be observed among the two groups (Figure 1a,c, representative images are shown). Quantitative analysis of the number of osteoclasts within the ROI indicated no significant differences between the two groups (Figure 1d, Table 2).

### 3.3. TRAP

To further analyze the pharmacological inhibitory effects of IV and oral BPs on osteoclastogenic activity, the formation of multinucleated, mature osteoclasts positive for the osteoclast marker TRAP was analyzed. TRAP-positive osteoclasts could be identified via the characteristic presence of three or more nuclei and red coloration of the whole cell body. As shown in Figure 1a,e, although TRAP-positive osteoclasts could be identified in the specimens from all groups, TRAP activity was significantly attenuated in the IV-BP group compared to that of the oral-BP group (Table 2, *p* < 0.001). This indicates a powerful suppression of osteoclastic resorption activity through the treatment of IV BPs.

### 3.4. RANKL/OPG

**Figure 2:**
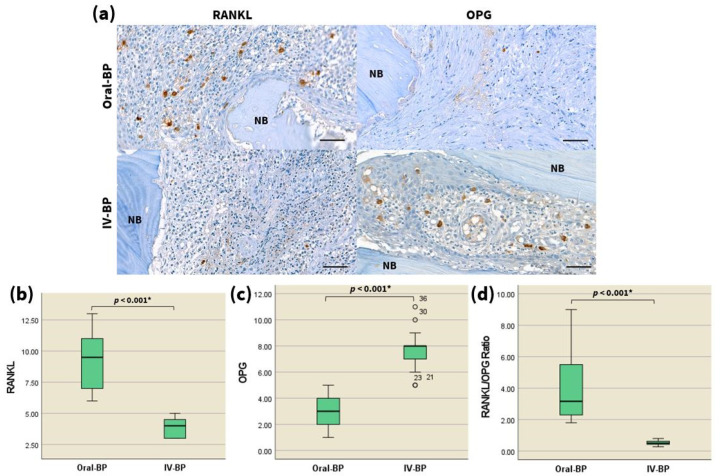
The RANKL/OPG ratio is decreased in the necrotic bones of IV BP-induced advanced-stage MRONJ patients. (**a**) High-magnification images (400×) of the immunohistochemistry analysis of RANKL (left) and OPG (right) expression in the specimens of the two patient groups as indicated. Scale bars = 50 μm. Statistical analysis of the number of RANKL-positive (**b**), and OPG-positive (**c**) cells, and the RANKL/OPG ratio (**d**), via the Mann–Whitney U test (*n* = 20 per group). ° marks statistical outliers. For detailed data, see Table 2. * *p* < 0.001.

We also sought to evaluate the changes in the bone turnover of MRONJ patients after IV and oral bisphosphonate treatment at cellular and molecular levels. To achieve this, the differences in the immunohistochemical reactivity for RANKL and OPG were compared between the two groups. The expression of RANKL (Figure 2a,b, representative images are shown) was significantly decreased in the bones of IV-BP patients compared with the oral-BP patients (*p* < 0.001), whereas a higher number of OPG-positive cells (Figure 2a,c, representative images are shown) was indicated among the former group (*p* < 0.001). Consequently, the RANKL/OPG ratio (Figure 2d) was markedly decreased in the histologic specimens of MRONJ patients with a history of IV BP administration compared with that of the respective oral-BP subjects (Table 2, *p* < 0.001). These findings suggested that IV BPs could downregulate the RANKL/OPG ratio to a significantly higher level than that induced via oral BPs, indicating that the relative deficiency of the RANKL/OPG ratio in patients taking intravenous BPs may potentially lead to the development of advanced stages of MRONJ.

### 3.5. Kcnn4

**Figure 3:**
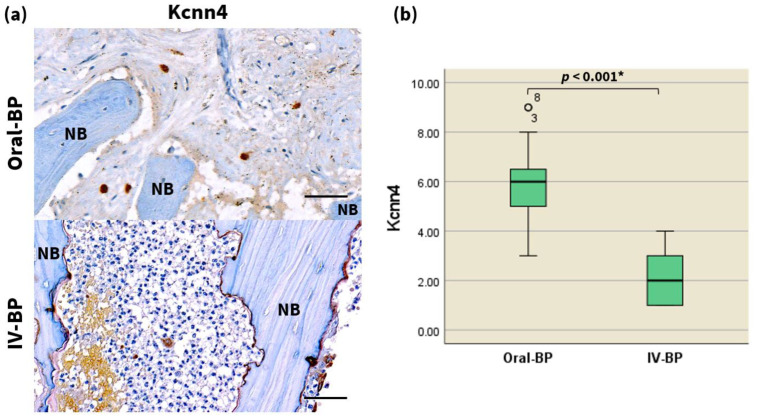
Kcnn4 expression is decreased in the necrotic bones of IV BP-induced advanced-stage MRONJ patients. (**a**) High-magnification images (400×) of the immunohistochemistry analysis of Kcnn4 expression in the specimens of the two patient groups as indicated. Scale bars = 50 μm. Statistical analysis of the number of Kcnn4-positive cells (**b**), via the Mann–Whitney U test (*n* = 20 per group). ° marks statistical outliers. For detailed data, see Table 2. * *p* < 0.001.

To investigate the regulation of calcium-activated potassium ion channels associated with osteoclast differentiation following IV and oral BP treatment, we examined the protein expression levels of Kcnn4. Immunohistochemistry demonstrated the presence of Kcnn4-positive cells in both groups, as characterized by the brown staining predominantly localized in the nucleus and cytoplasm. The level of Kcnn4 was revealed to be significantly downregulated in the tissues of IV-BP subjects compared with the oral-BP group counterparts (Figure 3, Table 2, *p* < 0.001). Taken together, these data suggest that the suppressed osteoclastogenic activity observed among IV-BP patients may also be related to the significant inhibition of KCa3.1 currents that are essential for RANKL-induced Ca^2+^ responses.

## 4. Discussion

Bisphosphonates are very potent inhibitors of osteoclast-mediated bone resorption [13,52] and are thus the most frequently used class of antiresorptives for the treatment of osteoporosis worldwide [1,12]. Although oral bisphosphonates are the mainstay of treatment for osteoporosis, they cannot be prescribed for some populations due to gastrointestinal intolerance or difficulty in complying with the dosing requirements [9,53,54]. In such patients, the intravenous injection of ibandronate [54,55,56,57], pamidronate [52,53,58], or zoledronate [59,60] has been shown to be an efficacious, well-tolerated, and convenient alternative to oral bisphosphonate therapy. However, despite the frequent prescription of intravenous bisphosphonates for the treatment and prevention of osteoporosis, to date all accumulated knowledge regarding the effect of BP administration route on the risk and prognosis of MRONJ derives from studies of patients who were prescribed IV BPs for the treatment of malignant conditions [9,12,39,45,46,47,48,49,50,51]. For the treatment of malignancies, 10-to-12-fold higher doses of IV BPs are prescribed than those used for osteoporosis [61,62,63,64]. Hence, the possibility of the dose-dependent effect of BPs on MRONJ progression, along with other contributing factors on poor prognosis such as insufficient nutrition and immune status, the presence of comorbidities, and exposure to other potentially toxic agents, cannot be ruled out [12,39,65]. Therefore, the higher risk and morbidity observed among the IV BP-induced MRONJ population of the previous reports cannot be attributed to the administration route alone, and the salient relationship between this factor and MRONJ prognosis remains unclear.

The present study hence describes a new relationship between the administration method of bisphosphonates and MRONJ progression. Our results demonstrate that bisphosphonates provided via the parenteral route exert a stronger suppressive effect on the expression levels of osteoclastic markers such as TRAP, RANKL/OPG ratio, and Kcnn4 compared to those administered per os, regardless of the medication dosage prescribed. To our knowledge, this is the first contribution in the literature to compare the anticipated effect of these two BP administration routes on the development of MRONJ in a context where all subjects were prescribed similar doses for the treatment of osteoporosis.

### 4.1. Anomalies of Osteoclasts in MRONJ Specimens

The inhibitory effect on osteoclastic bone resorption of bisphosphonates arises from a mechanism in which their impairment of the mevalonic acid sterol pathway results in subsequent disruption of the small GTPase signaling proteins required for the formation of the osteoclast cytoskeleton and ruffled border [11,14,16]. Previous experimental evidence obtained both in vivo and in vitro suggested that although this leads to profound attenuation of the individual resorption capabilities of osteoclasts following their detachment from bone surface and degeneration of the cytoskeleton, it does not necessarily result in the decrease of total osteoclasts number [2,14,16,66,67]. It has also been noted in prior studies that nitrogen-containing bisphosphonates prolong the lifespan of dysfunctional osteoclasts by diminishing the calcium signals required for their apoptosis, and thus permits excessive fusion of osteoclasts with nearby mononuclear progenitors, which would explain the increased size and nuclearity of osteoclasts observed in MRONJ bones [2,66]. Indeed, our cultures of the necrotic lesions from all subjects consistently expressed giant, hypernucleated osteoclasts that were detached from bone surface with pyknotic nuclei. Although not statistically significant, the larger osteoclasts observed among the IV-BP subjects may thus indicate the enhanced adverse effects of IV BPs on osteoclasts compared to those of oral BPs. It is also interesting to note here that while the total number, nuclearity, and size of osteoclasts observed did not differ significantly between the two groups, the osteoclasts of the IV-BP group were severely dysfunctional with respect to bone resorption, as assessed by the TRAP staining assay. Taken together, it can be concluded that the intravenous route of BP results in a more severe inhibition of the individual activity of osteoclasts, although they may not necessarily lead to increased apoptosis and subsequent decrease in the total osteoclast number.

### 4.2. Differences in Expression of Osteoclast-Related Markers—RANKL/OPG

To fully comprehend the mechanism through which the osteoclastic activity is inhibited by intravenous bisphosphonates, assays of well-known molecular bone markers were also performed. The maintenance of a skeletal homeostasis requires that the adequate number of mature osteoblasts and osteoclasts be produced to meet the requirements of the bone remodeling process. The binding of RANKL, a type II homotrimeric transmembrane protein that belongs to the tumor necrosis factor (TNF) superfamily [18,20,25], to its cognate receptor RANK results in the expression of NFATc1, the master transcription factor for osteoclastogenesis [11,17,21]. OPG acts as a decoy receptor by sequestering RANKL and inhibiting RANK signaling, thus exerting an antagonistic effect on osteoclast-mediated bone resorption [20,24,25,68,69]. The RANKL/RANK/OPG signaling pathway of osteoclasts thus performs a central role in determining bone mass by regulating the balance between osteoclastic bone resorption and osteoblastic bone formation [24,25,26].

Several previous studies of bisphosphonate-treated human and murine jaw bones have revealed significant downregulation of the RANKL/OPG ratio following bisphosphonate administration [4,53,70,71,72]. The subsequent bone turnover suppression following BP-induced RANKL/OPG downregulation can ultimately result in bone remodeling failure and the development of osteonecrosis after dentoalveolar surgery. The RANKL/OPG ratio is therefore an important biomarker for assessing the severity of MRONJ lesions [4,53,70]. The results of the present study demonstrate that intravenous bisphosphonates inhibited the RANKL/OPG ratio at a greater level than their oral counterparts, and that these findings are linked with decreased osteoclast activity, as shown by the TRAP analysis. Taken together with earlier observations that a decreased RANKL/OPG ratio leads to suppressed bone turnover in MRONJ patients [4,53,70], our results provide compelling evidence that the administration route of bisphosphonates is an essential determinant of the severity of the disease.

### 4.3. Differences in Expression of Kcnn4

Because the activation of most NFAT transcription factor family members is regulated by calcium/calmodulin signaling, the RANKL-dependent osteoclast differentiation via the activation of NFATc1 is critically dependent on Ca^2+^ signals [17,21]. Recent microarray analyses using murine cultures have corroborated the importance of Kcnn4 as the sole Ca^2+^ regulator of RANKL-activated multinucleation in both osteoclasts and multinucleated giant cells [15,17,28,31]. Modulation of the Kcnn4 channel represent a potentially therapeutic approach for skeletal pathologic conditions such as multiple sclerosis, inflammatory arthritis, and osteoporosis [15,28,31]. Inhibited Kcnn4 levels have also been confirmed as part of the pharmacological mechanism of ibandronate [15,17].

Despite the recent advances in our knowledge about the mechanisms by which Kcnn4 modulates the activity of osteoclasts in pathologic conditions, the key question regarding its anticipated role in the pathogenesis of MRONJ, remains unanswered. Because the mechanism of bisphosphonates involves the suppression of Kcnn4 [17], we postulated that the downregulation of this protein may also be important for the progression of bisphosphonate-induced osteonecrosis. To test this hypothesis, we analyzed the cellular expression of Kcnn4 in patients with history of IV and PO bisphosphonate treatment through immunohistochemistry. The results of our study established the significant inhibition of Kcnn4 in the IV BP-induced MRONJ patient group, consistent with other immunohistochemical results of our study.

## 5. Conclusions

The present findings have demonstrated differences in TRAP activity, RANKL, OPG, and Kcnn4 immunodetection among MRONJ lesions induced via different bisphosphonate administration routes at similar doses. The higher OPG and lower TRAP, RANKL, and Kcnn4 expression levels that were detected in the bones of IV-BP derived MRONJ patients can be correlated with enhanced suppression of osteoclast resorbing activity and subsequent osteonecrosis exacerbation. We thus show that intravenous injection of bisphosphonates aggravates the progression of the condition. In conclusion, although our findings need to be confirmed in a larger series, the present study suggests the potential of bisphosphonate administration route as an independent prognostic factor for advanced-stage MRONJ patients, regardless of the dosage or indication for which the bisphosphonate was prescribed.

## Figures and Tables

**Table 1:** Baseline characteristics of patients selected for the study (*N* = 20).

Group	Age	Gender	BP Type	Dose	BP Duration (Months)
Oral-BP	73	F	Ibandronate	150 mg/month	107
Oral-BP	84	M	Alendronate	70 mg/week	2
Oral-BP	73	F	Alendronate	35 mg/week	7
Oral-BP	79	F	Ibandronate	150 mg/month	20
Oral-BP	80	F	Risedronate	35 mg/week	7
Oral-BP	78	F	Alendronate	70 mg/week	20
Oral-BP	81	F	Ibandronate	150 mg/month	20
Oral-BP	80	F	Ibandronate	150 mg/month	5
Oral-BP	72	F	Risedronate	35 mg/week	5
Oral-BP	77	F	Alendronate	70 mg/week	3
IV-BP	84	F	Ibandronate Hydrate	3 mg/3 months	1.5
IV-BP	75	F	Zoledronate Hydrate	5 mg/year	3
IV-BP	79	M	Ibandronate Hydrate	3 mg/3 months	1
IV-BP	81	F	Ibandronate Hydrate	3 mg/3 months	2
IV-BP	79	F	Ibandronate Hydrate	3 mg/3 months	4
IV-BP	73	F	Ibandronate Hydrate	3 mg/3 months	3
IV-BP	74	F	Zoledronate Hydrate	5 mg/year	3
IV-BP	84	F	Zoledronate Hydrate	5 mg/year	2
IV-BP	80	F	Zoledronate Hydrate	5 mg/year	3
IV-BP	76	F	Ibandronate Hydrate	3 mg/3 months	3

BP, bisphosphonate; F, female; M, male; IV, intravenous.

**Table 2:** H&E, TRAP, and IHC analysis of the specimens retrieved from the Oral-BP, IV-BP groups.

	Group	Minimum	Maximum	Average	Median	IQR	SD	*p*-Value ^1^
H&E Staining								0.09
Osteoclast diameter (μm)	Oral-BP	2	8	4.04	4	2	0.296	
	IV-BP	2	11	5.26	5	3	0.496	
Nuclearity of osteoclasts (nuclei/osteoclast)	Oral-BP	15.4	35.7	23.285	23.2	6.4	1.046	0.063
	IV-BP	17.1	42.5	27.083	25.7	10.6	1.461	
Osteoclasts per ROI	Oral-BP	1	5	2.17	2	2	0.366	0.32
	IV-BP	1	3	1.62	1	1	0.213	
TRAP staining								**<0.001 ^2^**
TRAP+ osteoclasts per ROI	Oral-BP	6	10	8.25	8.	1	0.204	
	IV-BP	2	5	3.15	3	2	0.221	
Immunohistochemistry								**<0.001 ^2^**
RANKL+ cells per ROI	Oral-BP	6	13	9.25	9.5	4	0.502	
	IV-BP	3	5	3.88	4	2	0.202	
OPG+ cells per ROI	Oral-BP	1	5	2.9	3	2	0.307	**<0.001 ^2^**
	IV-BP	5	11	7.65	8	2	0.373	
RANKL/OPG ratio	Oral-BP	1.8	9	3.899	3.167	3.35	0.438	**<0.001 ^2^**
	IV-BP	0.27	0.8	0.523	0.5	0.2	0.04	
Kcnn4+ cells per ROI	Oral-BP	3	10	6	6	2	0.447	**<0.001 ^2^**
	IV-BP	0	4	1.9	2	2	0.261	

IQR, interquartile range; SD, standard deviation; H&E, hematoxylin and eosin; ROI, region of interest. ^1^ *p*-Values were obtained via the Mann–Whitney U test; ^2^ bold values considered as significant (*p* < 0.05).

## Data Availability

The data presented in this study are available on request from the corresponding author.

## Abbreviations

BP, bisphosphonates; DAB, diaminobenzidine; H&E, hematoxylin–eosin; IHC, immunohistochemistry; IQR, interquartile range; IV, intravenous; Kcnn4, potassium calcium-activated channel subfamily N member 4; MRONJ, medication-related osteonecrosis of the jaw; NB, necrotic bone; OPG, osteoprotegerin; RANK, receptor activator of NF-κB; RANKL, receptor activator of NF-κB ligand; ROI, region of interest; SD, standard deviation; SREs, skeletal related events; TRAP, tartrate-resistant acid phosphatase.
